# Moderate Alcohol Use and Cardiovascular Disease from Mendelian Randomization

**DOI:** 10.1371/journal.pone.0068054

**Published:** 2013-07-16

**Authors:** Shiu Lun Au Yeung, Chaoqiang Jiang, Kar Keung Cheng, Benjamin J. Cowling, Bin Liu, Weisen Zhang, Tai Hing Lam, Gabriel M. Leung, C. Mary Schooling

**Affiliations:** 1 Lifestyle and Lifecourse Epidemiology Group, School of Public Health, Li Ka Shing Faculty of Medicine, The University of Hong Kong, Hong Kong SAR, China; 2 Guangzhou Number 12 Hospital, Guangzhou, China; 3 Department of Public Health and Epidemiology, University of Birmingham, Birmingham, United Kingdom; 4 School of Public Health, City University of New York, New York, United States of America; Sapienza University of Rome, Italy

## Abstract

**Background:**

Observational studies show moderate alcohol use negatively associated with ischemic heart disease (IHD) and cardiovascular disease (CVD). However, healthier attributes among moderate users compared to never users may confound the apparent association. A potentially less biased way to examine the association is Mendelian randomization, using alcohol metabolizing genes which influence alcohol use.

**Methods:**

We used instrumental variable analysis with aldehyde dehydrogenase 2 (*ALDH2*) genotypes (AA/GA/GG) as instrumental variables for alcohol use to examine the association of alcohol use (10 g ethanol/day) with CVD risk factors (blood pressure, lipids and glucose) and morbidity (self-reported IHD and CVD) among men in the Guangzhou Biobank Cohort Study.

**Results:**

*ALDH2* genotypes were a credible instrument for alcohol use (F-statistic 74.6). Alcohol was positively associated with HDL-cholesterol (0.05 mmol/L per alcohol unit, 95% confidence interval (CI) 0.02 to 0.08) and diastolic blood pressure (1.15 mmHg, 95% CI 0.23 to 2.07) but not with systolic blood pressure (1.00 mmHg, 95% CI -0.74 to 2.74), LDL-cholesterol (0.03 mmol/L, 95% CI -0.03 to 0.08), log transformed triglycerides (0.03 mmol/L, 95% CI -0.01 to 0.08) or log transformed fasting glucose (0.01 mmol/L, 95% CI -0.006 to 0.03), self-reported CVD (odds ratio (OR) 0.98, 95% CI 0.76 to 1.27) or self-reported IHD (OR 1.10, 95% CI 0.83 to 1.45).

**Conclusion:**

Low to moderate alcohol use among men had the expected effects on most CVD risk factors but not fasting glucose. Larger studies are needed to confirm the null associations with IHD, CVD and fasting glucose.

## Introduction

In observational studies alcohol usually has a U or J shaped association with health, such that moderate alcohol use (1–2 drinks per day) is negatively associated with ischemic heart disease (IHD) [1] ischemic stroke [2] and diabetes [3]. Correspondingly, some public health advice implies that moderate alcohol use is protective against cardiovascular disease (CVD) and diabetes [4]. However, observational studies are vulnerable to biases from residual confounding, due to unmeasured systemic differences between moderate alcohol and other alcohol users [5], from over-adjustment due to an imperfect understanding of the underlying causal pathways and from reverse causality, thus such studies may not provide a sound basis for causal inference. Notably, the benefits of moderate alcohol use are not always evident in studies from the majority of the global population outside western settings [6]–[8] where moderate alcohol use is less commonly used as a social lubricant.

Evidence from randomized controlled trials (RCTs) suggests that alcohol monotonically increases blood pressure [9], [10], high density lipoprotein (HDL)-cholesterol [11] and triglycerides [11], whilst the evidence concerning diabetes and glucose metabolism is more limited [12], [13]. There have been no large-scale RCTs of moderate alcohol use and cardiovascular disease most likely due to ethical and practical issues. A Mendelian randomization study provides an alternative approach to establish the causal role of moderate alcohol use in a suitable population where a genetic variant affects alcohol metabolism and thereby alcohol use. Genetic variants in the aldehyde dehydrogenase 2 (*ALDH2*) gene do affect alcohol metabolism [14] and have been proposed as suitable predictors of alcohol use for use in Mendelian randomization studies [15]. Mendelian randomization studies can be thought of in three ways. First, the genotypes can serve as proxies of the exposure [15], which is particularly useful if the relevant exposure has not been or cannot be measured. Second, Mendelian randomization can be thought of as triangulation of genotype, exposure and outcome, akin to mediation, which is an intuitive way of thinking of instrumental variable analysis. Third, Mendelian randomization can be thought of as instrumental variable analysis with genetic instruments, which was used in this study [16], [17], because instrumental variable analysis is a well-established statistical technique with known statistical assumptions and properties. *ALDH2* alleles are randomly allocated to at conception. People with inactive *ALDH2* alleles flush and feel discomfort following alcohol use because of acetaldehyde exposure [18]. Genetically determined alcohol use resembles random allocation in RCTs, and provides a potentially less biased way to assess the effect of moderate alcohol use on health [15]. People, with inactive *ALDH2* alleles, and correspondingly lower alcohol consumption, have been observed to have lower blood pressure [19] and lower HDL-cholesterol [20], consistent with evidence from RCTs. However these studies lacked information on alcohol use and used *ALDH2* genotypes as proxies of alcohol consumption, rather than using instrumental variable analysis to estimate the effect of alcohol use on CVD risk factors.

Southern Chinese men are uniquely suitable for a Mendelian randomization study of alcohol use with *ALDH2* genotype as a genetic instrument. Inactive alleles of *ALDH2* are common in East Asia. In our study of Southern Chinese *ALDH2* alleles are consistent with Hardy Weinberg equilibrium as required for Mendelian randomization [21]. *ALDH2* was also strongly associated with alcohol use among men but not associated with potential confounders [21]. We used Mendelian randomization to assess whether moderate alcohol use was protective against self-reported CVD, self-reported IHD or major CVD risk factors among men from the Guangzhou Biobank Cohort Study. We also assessed the same associations using multivariable regression adjusted for confounders in an observational study design for comparison.

## Methods

### Ethics statement

The Guangzhou Medical Ethics Committee of the Chinese Medical Association approved the study and all participants gave written, informed consent before participation.

### Participants

The Guangzhou Biobank Cohort Study (GBCS) is a collaboration between the Guangzhou No.12 Hospital and the Universities of Hong Kong and Birmingham [22]. Recruitment of participants draws from “The Guangzhou Health and Happiness Association for the Respectable Elders (GHHARE)”, a community social and welfare association unofficially aligned with the municipal government where membership is open to anyone aged 50 years or older for a monthly, nominal fee of 4 Yuan (50 US cents). There were three recruitment phases. Recruitment for phase 1 took place from September 2003 to November 2004, for phase 2 from April 2005 to May 2006, and for phase 3 from September 2006 to January 2008. Follow-up of the participants started in 2008. Approximately 7% of permanent Guangzhou residents aged 50 years or more are members of GHHARE, of whom 33% enrolled for phases 1, 2 or 3 recruitment and were included if they were capable of consenting, ambulatory, and not receiving treatment modalities that, if omitted, may result in immediate life-threatening risk, such as chemotherapy or radiotherapy for cancer, or dialysis for renal failure. Participants in GBCS are ethnic Chinese largely from southern China. Participants underwent a detailed interview and physical examination at baseline recruitment, including medical history and report of doctor diagnosed conditions. Participants were asked about doctor diagnosed CVD, and if applicable the type of CVD, such as IHD or stroke. The methods of measurement have previously been reported [22]. Alcohol use was recorded in terms of frequency, type of beverage and usual amount per occasion. We recorded seated blood pressure as the average of the last two or three measurements, using the Omron 705CP sphygmomanometer (Omron Corp., Kyoto, Japan). Fasting low density lipoprotein (LDL)-cholesterol, high density lipoprotein (HDL)-cholesterol, triglycerides, and glucose were determined with a Shimadzu CL-8000 clinical chemical analyzer (Shimadzu Corp, Kyoto, Japan) in the hospital laboratory.

### DNA extraction and SNP analysis

As previously described [21], biological samples for DNA extraction used in the present study were obtained in GBCS phase 3 at recruitment and in phases 1 and 2 at follow-up. Of the 8,450 men in all three GBCS phases, 5,606 had bio-materials suitable for DNA extraction. DNA was extracted at Guangzhou No. 12 Hospital either from fresh blood using a standard phenol-chloroform extraction procedure and stored at −80°C or from blood or buffy coat previously stored at −80°C using a standard magnetic bead extraction procedure. There were 4,987 men with viable DNA for genotyping. Genotyping of SNP rs671 to identify *ALDH2* genotypes (AA, GA or GG) was performed using the MassARRAY system (Sequenom, San Diego, CA, USA) and the iPLEX assay at a commercial company (Beijing CapitalBio Corporation, Beijing, China).

### Alcohol use

The main exposure was continuous alcohol units (10 gram (g) ethanol per day) based on total alcohol consumption obtained from the frequency, quantity and type recorded at recruitment [21]. Specifically, we asked the participants how often they drank alcohol (once or twice per year, once every couple of months, <1 day/ week, 1–2 days/week, 3–4 days/week, 5–6 days/week, daily or almost every day), the type of alcohol usually consumed, and how much of each type of alcohol (beer, western table wine, spirits, Chinese rice wine or Chinese rice wine (high strength)) usually consumed per occasion, from which we calculated units per day. Infeasible amounts (>30 alcohol units per day) were excluded [21]. Former alcohol users were included as non-drinkers because former alcohol users may have abstained from alcohol because of poor health unrelated to former alcohol use; excluding them could create a bias. Many former users reported previously infrequent alcohol use, i.e., once or twice a year.

### Outcomes

The outcomes were doctor diagnosed self-reported CVD, which included IHD, stroke/transient ischemic attack, angina, myocardial infarction, peripheral vascular disease, valvular heart disease and rheumatic heart disease, self-reported IHD, which included IHD, angina and myocardial infarction, and biological CVD risk factors, i.e., systolic blood pressure, diastolic blood pressure, HDL-cholesterol, LDL-cholesterol and fasting plasma glucose, reflecting biological risk factors in the Framingham equation. We also included a related risk factor, triglycerides, because alcohol use has been positively associated with triglycerides in RCTs [11]. Since triglycerides and fasting glucose were not normally distributed, they were log transformed.

### Statistical analysis

**Figure 1:**
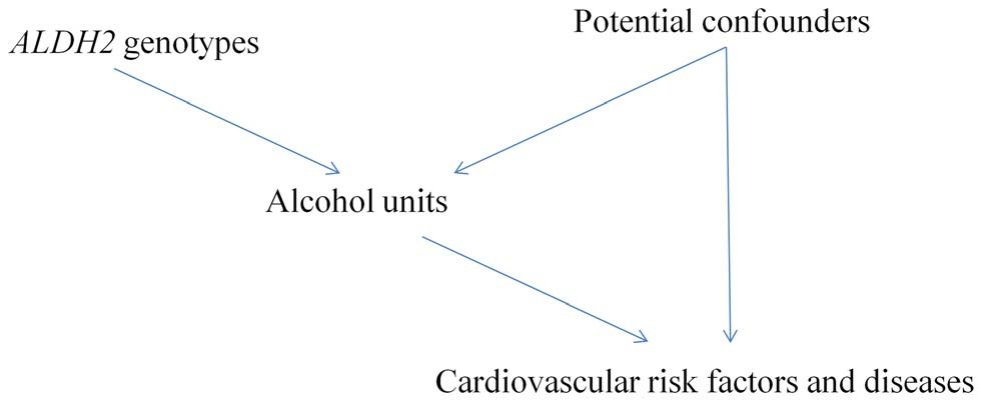
Directed acyclic graph showing the framework of Mendelian randomization analyses in this study.

We used analysis of variance (ANOVA) to assess the associations of ALDH2 genotypes with alcohol consumption, blood pressure and body mass index. We used chi-square tests to assess whether *ALDH2* genotypes were associated with potential confounders, such as socioeconomic position, lifestyle, and medication use. We used instrumental variable analysis (2 stage least squares (2SLS)) with *ALDH*2 genotype categories as an instrumental variable, because there was a non-linear association with alcohol consumption, to obtain estimates of alcohol use on CVD risk factors, from which we reported β coefficients with 95% confidence intervals. Such β coefficients represent the effect of one unit of alcohol on the CVD risk factor. This instrumental variable analysis obtains the effect of alcohol use on each CVD risk factor from the association of *ALDH2* with the CVD risk factor divided by the association of *ALDH2* with alcohol use, i.e., by using the relations between genotype, alcohol use and CVD risk factors. For CVD morbidity, we used instrumental variable probit regression analysis to obtain the probit coefficient, which we multiplied by 1.6 to obtain the approximate logarithm of the odds ratio [23]. We did not adjust for confounders in 2SLS or the instrumental variable probit regression because *ALDH2* genotypes randomly allocated at conception cannot be confounded by age or subsequent socio-economic position, lifestyle or medication use. Figure 1 depicts the directed acyclic graph for the Mendelian randomization analyses, i.e., instrumental variable analysis with genetic instruments, used in this study.

For comparison, we also present the adjusted associations of alcohol units with the outcomes under multivariable regression models in an observational design. We adjusted these multivariable regression models for age, education, physical activity, smoking status and additionally adjusted analysis of CVD risk factors for use of appropriate medication.

### Sensitivity analyses

We repeated the analysis excluding former users in a sensitivity analysis. Instrumental variable analysis assumes linear association between alcohol and the CVD. To account for any potential U shaped relation of alcohol use with CVD, we also repeated the analysis excluding heavy users (weekly drinking >210 g ethanol/week) because alcohol use below this level is usually more monotonic, and so can be modeled as linear.

All statistical analyses were conducted using Stata version 10.1 (StataCorp LP, College Station, TX).

## Results

Of the 4,987 men with viable DNA, 4,867 had complete information on *ALDH2* genotypes, alcohol use and at least one outcome. Table 1 shows that men with two active *ALDH2* alleles on average consumed ten times as much alcohol per day (0.9 units) as men with two inactive alleles (0.09 units). Fifty one percent (51%) of men were never users. Among current users, the mean alcohol units consumed was 1.30 (standard deviation 2.88) while the main alcohol beverage type used was Chinese rice wine (64%). Relatively few men reported IHD (4.8%) or CVD (6.4%) consistent with mortality from these causes in China [24]. *ALDH2* satisfies the assumptions for being a credible instrument for alcohol use, including little direct association with the outcomes considered [25], and little association with potential confounders, such as age, socioeconomic positions and lifestyle (Table 1).

**Table 1:** Alcohol consumption and socio-demographic characteristics by *ALDH2* genotype among men from the Guangzhou Biobank Cohort Study (2003–8).

		*ALDH2* genotype (from rs671)			
		Two inactive alleles (AA)	One inactive allele (AG/GA)	No inactive alleles (GG)	§P value
Alcohol units (10g	n	416	2,023	2,428	
ethanol) per day	mean (SD)	0.09 (0.79)	0.24 (1.22)	0.90 (2.52)	<0.001
Age group (%) years	n, years	417	2,053	2,457	
	50–54	11.0	10.0	9.2	0.41
	55–59	20.9	20.9	21.2	
	60–64	25.9	23.9	26.3	
	65–69	19.7	23.8	23.4	
	70–74	16.3	15.7	14.6	
	75–79	5.5	4.2	3.7	
	80+	0.7	1.5	1.4	
Education (%)	n	417	2,051	2,455	
	Less than primary	2.6	2.3	2.3	0.63
	Primary	24.7	27.3	26.2	
	Junior middle	29.0	30.3	31.1	
	Senior middle	27.1	25.1	23.5	
	Junior college	10.3	8.5	9.2	
	College	6.2	6.5	7.7	
Smoking status (%)	n	416	2,045	2,444	
	Never	41.1	40.4	40.1	0.88
	Former	29.3	27.7	27.8	
	Current	29.6	31.9	32.2	
Physical activity	n	417	2,053	2,457	
(IPAQ) (%)	Inactive	9.1	8.5	8.1	0.23
	Minimally active	36.9	38.8	41.6	
	†HEPA active	54.0	52.7	50.3	
Antihypertensive	n	416	2,045	2,451	
drugs (%)	Current user	19.5	18.7	20.2	0.49
Lipid modifying	n	417	2,052	2,453	
drugs (%)	Current user	5.5	5.4	6.3	0.44
drugs (%)	Current user	6.2	6.2	6.6	0.87
Systolic blood	n	416	2,046	2,449	
pressure (mmHg)	mean (SD)	131.2 (19.3)	132.7 (21.1)	133.0 (21.7)	0.31
Diastolic blood	n	415	2,046	2,446	
pressure (mmHg)	mean (SD)	75.3 (10.4)	75.8 (10.9)	76.5 (11.4)	0.05
Body Mass	n	416	2,048	2,448	
Index (kg/m^2^)	mean (SD)	23.5 (3.0)	23.5 (3.1)	23.5 (3.2)	0.84

§ P-value from ANOVA for continuous variables and from a χ^2^ test for categorical variables, 2 sided.

† HEPA: health-enhancing physical activity (i.e., vigorous activity at least 3 days a week achieving at least 1,500 metabolic equivalent (MET) minutes per week or activity on 7 days of the week, achieving at least 3,000 MET minutes per week; IPAQ: International Physical Activity Questionnaire.

The F-statistic from the first stage of the instrumental variable analysis was 74.6 (r^2^  = 0.03), suggesting that weak instrument bias was unlikely. There was no evidence that the association of *ALDH2* genotypes with alcohol units varied with any of the outcomes considered. Table 2 shows that in the Mendelian randomization analysis alcohol was positively associated with diastolic blood pressure, and HDL-cholesterol. Alcohol was not associated with systolic blood pressure, LDL-cholesterol, log transformed triglycerides, log transformed fasting plasma glucose, self-reported IHD or self-reported CVD.

**Table 2:** Mendelian randomization estimates, obtained from instrumental variable analysis using 2SLS and probit regression, and multivariable linear and probit regression estimates of the association of alcohol use (1 unit) with CVD risk factors and morbidity.

	Mendelian randomization Instrumental variable analysis				†Observational Multivariable regression			
	n	§β	95% CI	p value	n	§β	95% CI	p value
Systolic blood pressure (mmHg)	4,853	1.00	−0.74 to 2.74	0.26	4,847	0.84	0.55 to 1.12	<0.001
Diastolic blood pressure (mmHg)	4,849	1.15	0.23 to 2.07	0.01	4,843	0.49	0.34 to 0.65	<0.001
Log transformed triglycerides (mmol/L)	4,844	0.03	−0.01 to 0.08	0.13	4,836	0.006	−0.002 to 0.01	0.12
HDL-cholesterol (mmol/L)	4,843	0.05	0.02 to 0.08	0.003	4,835	0.01	0.008 to 0.02	<0.001
LDL-cholesterol (mmol/L)	4,838	0.03	−0.03 to 0.08	0.31	4,830	0.009	0.00 to 0.02	0.05
Log transformed fasting glucose (mmol/L)	4,837	0.01	−0.006 to 0.03	0.23	4,830	0.005	0.002 to 0.007	<0.001

	n	Odds ratio	95% CI	p value	n	Odds ratio	95% CI	p value
Self-reported CVD	4,867	0.98	0.76 to 1.27	0.90	4,864	0.99	0.95 to 1.04	0.82
Self-reported IHD	4,867	1.10	0.83 to 1.45	0.50	4,864	0.99	0.94 to 1.05	0.77

† adjusted for age, education, physical activity, smoking and use of appropriate medication (CVD risk factors only).

§ per alcohol unit change (10 grams ethanol/day).

¶ Odds ratio is approximated by the antilogarithm of (1.6× probit coefficient).

Most of the observational estimates from multivariable regression were in the same direction as, but smaller than, the Mendelian randomization estimates. Alcohol was also positively associated with systolic blood pressure, LDL-cholesterol, and fasting glucose, but was not associated with self-reported CVD or self-reported IHD. Associations were similar when former users were excluded (Table 3) although estimates were larger when heavy users were excluded (Table 4). Table 5 shows analysis stratified by age, because genetic associations may be less evident at older ages when aging and ill-health may have a greater impact on CVD and its risk factors. The effects of alcohol were more evident among the younger men.

**Table 3:** Mendelian randomization estimates, obtained from instrumental variable analysis using 2SLS and probit regression, and multivariable linear and probit regression estimates of the association of alcohol use (1 unit) with CVD risk factors and morbidity, excluding former users.

	Mendelian randomization Instrumental variable analysis					†Observational Multivariable regression			
	n	§β	95% CI		p value	n	§β	95% CI	p value
Systolic blood pressure (mmHg)	4,559	1.14	−0.50 to 2.77		0.17	4,553	0.84	0.55 to 1.12	<0.001
								
Diastolic blood pressure (mmHg)	4,557	1.37	0.50 to 2.24		0.002	4,551	0.48	0.33 to 0.64	<0.001
									
Log transformed triglycerides (mmol/L)	4,551	0.03	−0.008 to 0.08		0.11	4,543	0.006	−0.001 to 0.01	0.11
									
HDL-cholesterol (mmol/L)	4,550	0.05	0.02 to 0.08		0.001	4,542	0.01	0.008 to 0.02	<0.001
									
LDL-cholesterol (mmol/L)	4,547	0.04	−0.01 to 0.09		0.14	4,539	0.01	0.00 to 0.02	0.04
									
Log transformed fasting glucose (mmol/L)	4,544	0.005	−0.01 to 0.02		0.54	4,537	0.005	0.002 to 0.007	<0.001

	n	Odds ratio	95% CI	p value	n	Odds ratio	95% CI	p value
Self-reported CVD	4,572	0.95	0.74 to 1.21	0.68	4,569	1.00	0.95 to 1.04	0.87
								
Self-reported IHD	4,572	1.07	0.82 to 1.39	0.63	4,569	0.99	0.94 to 1.05	0.77

† adjusted for age, education, physical activity, smoking and use of appropriate medication (CVD risk factors only).

§ per alcohol unit change (10 grams ethanol/day).

¶ Odds ratio is approximated by the antilogarithm of (1.6× probit coefficient).

**Table 4:** Mendelian randomization estimates, obtained from instrumental variable analysis using 2SLS and probit regression, and multivariable linear and probit regression estimates of the association of alcohol use (1 unit) with CVD risk factors and morbidity, excluding heavy users.

	Mendelian randomization Instrumental variable analysis				†Observational Multivariable regression			
	n	§β	95% CI	p value	n	§β	95% CI	p value
Systolic blood pressure (mmHg)	4,568	1.30	−6.01 to 8.61	0.73	4,562	2.29	1.07 to 3.50	<0.001
Diastolic blood pressure (mmHg)	4,564	3.73	−0.11 to 7.57	0.06	4,558	1.74	1.08 to 2.39	<0.001
Log transformed triglycerides (mmol/L)	4,559	0.09	−0.10 to 0.28	0.34	4,552	0.00	−0.03 to 0.03	0.96
HDL-cholesterol (mmol/L)	4,558	0.16	0.03 to 0.29	0.01	4,551	0.05	0.03 to 0.08	<0.001
LDL-cholesterol (mmol/L)	4,553	0.09	−0.14 to 0.31	0.45	4,546	−0.01	−0.05 to 0.03	0.61
Log transformed fasting glucose (mmol/L)	4,552	0.02	−0.05 to 0.09	0.55	4,545	0.01	0.002 to 0.02	0.02

	n	Odds ratio	95% CI	p value	n	Odds ratio	95% CI	p value
Self-reported CVD	4,580	0.94	0.32 to 2.81	0.92	4,577	0.84	0.67 to 1.06	0.14
Self-reported IHD	4,580	1.67	0.53 to 5.23	0.38	4,577	0.80	0.62 to 1.05	0.10

† adjusted for age, education, physical activity, smoking and use of appropriate medication (CVD risk factors only).

§ per alcohol unit change (10 grams ethanol/day).

¶ Odds ratio is approximated by the antilogarithm of (1.6× probit coefficient).

**Table 5:** Mendelian randomization estimates, obtained from instrumental variable analysis using 2SLS and probit regression, and multivariable linear and probit regression estimates of the association of alcohol use (1 unit) with CVD risk factors and morbidity stratified by median age.

		Mendelian randomization Instrumental variable analysis				†Observational Multivariable regression			
Age group	CVD risk factor or morbidity	n	§β	95% CI	p value	n	§β	95% CI	p value
Age≦63.5	Systolic blood pressure (mmHg)	2,430	3.08	0.47 to 5.69	0.02	2,428	0.86	0.48 to 1.24	<0.001
Age >63.5	Systolic blood pressure (mmHg)	2,423	−0.57	−2.92 to 1.79	0.64	2,419	0.84	0.42 to 1.27	<0.001
Age≦63.5	Diastolic blood pressure (mmHg)	2,428	2.34	0.82 to 3.87	0.003	2,426	0.46	0.24 to 0.68	<0.001
Age >63.5	Diastolic blood pressure (mmHg)	2,421	0.06	−1.09 to 1.21	0.92	2,417	0.52	0.31 to 0.73	<0.001
Age≦63.5	Log transformed triglycerides (mmol/L)	2,424	0.04	−0.03 to 0.11	0.27	2,422	0.007	−0.004 to 0.02	0.22
Age >63.5	Log transformed triglycerides (mmol/L)	2,420	0.02	−0.03 to 0.08	0.39	2,414	0.005	−0.006 to 0.02	0.40
Age≦63.5	HDL-cholesterol (mmol/L)	2,423	0.07	0.02 to 0.11	0.005	2,421	0.02	0.01 to 0.03	<0.001
Age >63.5	HDL-cholesterol (mmol/L)	2,420	0.03	−0.01 to 0.07	0.16	2,414	0.01	0.002 to 0.02	0.02
Age≦63.5	LDL-cholesterol (mmol/L)	2,422	0.02	−0.06 to 0.10	0.60	2,420	0.004	−0.009 to 0.02	0.55
Age >63.5	LDL-cholesterol (mmol/L)	2,416	0.03	−0.04 to 0.10	0.40	2,410	0.02	0.002 to 0.03	0.03
Age≦63.5	Log transformed fasting glucose (mmol/L)	2,421	0.009	−0.02 to 0.03	0.46	2,420	0.005	0.001 to 0.008	0.01
Age >63.5	Log transformed fasting glucose (mmol/L)	2,416	0.01	−0.01 to 0.03	0.31	2,410	0.005	0.001 to 0.008	0.009

		n	¶Odds ratio	95% CI	p value	n	Odds ratio	95% CI	p value
Age≦63.5	Self-reported CVD	2,436	1.26	0.84 to 1.87	0.26	2,436	0.98	0.91 to 1.06	0.65
Age >63.5	Self-reported CVD	2,431	0.89	0.66 to 1.20	0.43	2,428	1.00	0.94 to 1.06	1.00
Age≦63.5	Self-reported IHD	2,436	1.38	0.93 to 2.05	0.11	2,436	1.00	0.92 to 1.08	0.96
Age >63.5	Self-reported IHD	2,431	0.99	0.71 to 1.39	0.97	2,428	0.99	0.92 to 1.06	0.69

† adjusted for age (continuous), education, physical activity, smoking and use of appropriate medication (CVD risk factors only).

§ per alcohol unit change (10 grams ethanol/day).

¶ Odds ratio is approximated by the antilogarithm of (1.6× probit coefficient).

## Discussion

Using a Mendelian randomization design, which utilizes genes randomly allocated at conception and is similar to the random allocation process of an RCT [16], in a suitable low to moderate drinking population of Southern Chinese men, we found low to moderate alcohol use led to higher diastolic blood pressure, and HDL-cholesterol, with no protective effect on fasting glucose, self-reported CVD or self-reported IHD. Our results are consistent with experimental and non-observational evidence showing that alcohol monotonically increases blood pressure [10], [19] and HDL-cholesterol [11] with no protective effect on glucose metabolism [26], although previous studies on glucose metabolism were not powered to detect a small effect [13]. Our study adds by confirming these findings in the first comprehensive Mendelian randomization analysis of the causal effects of moderate alcohol use on CVD, IHD and CVD risk factors. It also adds by showing, for the first time, that moderate alcohol use might not have a protective effect on IHD or CVD morbidity although larger studies are required to confirm these preliminary null findings.

A Mendelian randomization study design may provide a better means of generating an unbiased estimate, given systematic differences among alcohol users. However, limitations of this study remain. First, the effects of alcohol could vary by *ALDH2* genotype due to varying acetaldehyde exposure. However animal studies suggest acetaldehyde causes cardiovascular harm [27], which, if anything, would bias towards a protective effect of active *ALDH2* alleles and hence of alcohol. Second, Mendelian randomization studies in western populations, where beneficial effects of moderate alcohol use have most consistently been observed, might be most convincing. However, Caucasians lack *ALDH2* (rs671) polymorphisms and such studies would need to use *ADH* polymorphisms which have weaker associations with alcohol use in Chinese [28]. Third, the range of alcohol use was mainly low to moderate, beyond which our findings should not be extrapolated. A meta analysis of observational studies on alcohol use and cardiovascular outcomes suggest benefits of alcohol use was observed within this range [1]. Fourth, Mendelian randomization studies require large sample sizes for adequate power. Power calculation for Mendelian randomization analyses is not readily available. In the current Mendelian randomization analysis of ∼4,500 men, the r^2^ (squared correlation) for *ALDH2* genotypes with alcohol units was 0.03. Based on a recent simulation study, our analysis would be powered to detect effect sizes of at least 0.3 standard deviations [29], and analyses for CVD risk factors should be adequately powered. On the other hand, given the low prevalence of self-reported CVD and IHD in this study, the relevant analyses were likely to be underpowered and hence we only considered these analyses as preliminary. These analyses should be replicated in future studies with larger sample size. Fifth, this is not a population representative study, however, disease prevalence is similar to that in a representative urban Chinese population [22]. The generalizability of this study will be limited if amongst the men excluded from this study the effect of genetically determined alcohol use on CVD and its risk factors differed from that in the men included, which is unlikely. We did not have suitable bio-materials for all men from phases 1 and 2. However, *ALDH2* genotypes were not associated with recruitment phase. Sixth, grape wine was rarely consumed (∼10% of all current alcohol users) but the apparent benefits of alcohol appear to be independent of beverage types, where the rise in HDL-cholesterol is similar across beverage types [30]. Although the additional benefits from wine consumption could be due to resveratrol, or to confounding by socioeconomic position biasing the estimates from null [31], wine use is rare in Southern China, so our study is particularly suitable for establishing the effects of alcohol use. Seventh, we do not yet have prospective CVD events in this study. However, we used self-reported CVD and self-reported IHD, which have previously been shown to be associated with the metabolic syndrome in this study, indicating some validity of these self reports [32]. However, misclassification of CVD events arising from self-reports may have made it more difficult to detect any association resulting in the observed null associations. Eighth, Mendelian randomization studies assess the causal effects of exposure on outcome, Mendelian randomization studies do not address exactly how any causal effect is mediated. For example our null association of alcohol use with CVD is consistent with positive effects of alcohol on blood pressure being balanced out by positive effects on HDL, but could have occurred by other mechanisms, which is of etiological relevance and needs to be assessed. However, from a public health perspective the key question is the effect of alcohol regardless of how it is mediated. Ninth, Mendelian randomization studies require no direct association of the instrument with confounders or the outcomes, as well as homogenous associations and lack of population stratification [33]. We found no evidence of direct associations of the instrument with confounders in our previous Mendelian randomization study [21] or any direct genetic associations with the outcomes among a random sample of women who were mostly never/occasional users (data not shown). Our study was drawn from permanent residents of Guangzhou who are ethnically homogeneous. Nevertheless, as with any study, we cannot rule out all possible biases. In addition, Mendelian randomization requires additional assumptions and some of them are not always verifiable [33]. Tenth, we did not test for a curvilinear association of alcohol use with CVD or its risk factors as instrumental variable analysis assumes linearity between alcohol and the outcomes. On the other hand, in our setting alcohol use is generally low and in the range where the observed associations of alcohol use with CVD and its risk factors are usually linear. However, we presented an additional analysis excluding heavy users (Table 4), which gave a similar interpretation. In additional, although observational associations may be curvilinear, causal effects are more often linear, such that a dose-response relation is one of the Bradford Hill criteria for causality. Eleventh, genetic associations with health may be less evident at older ages, potentially creating a bias towards the null. In age-stratified analysis the effects of alcohol on CVD and its risk factors were most evident in the younger men (Table 5).

Despite these limitations, our findings for CVD risk factors are similar in direction and interpretation to those from non-observational studies. The Mendelian randomization estimates of the effect of one alcohol unit (10 g of ethanol per day) on diastolic blood pressure and HDL-cholesterol were higher than those reported from RCTs [10], [11]. Most RCTs of alcohol use concern free-living people in western populations. Any lapse in compliance would underestimate the effect of alcohol. Moreover, the effect of reducing from high alcohol consumption, as often examined [9], may not be the same as increasing from negligible or low alcohol consumption. Prior lifelong alcohol use may also compromise these inevitably short trials [10], [11]. Our estimates also had wide confidence intervals, which included estimates from intervention studies [9], [11]. However, it is possible that the differences in the estimates between our study and RCTs could be due to differences in the participants studied in these designs or violations of the instrumental variable assumptions [33], although we have established the credibility of *ALDH2* as an instrumental variable for alcohol use [25], or possible over-estimation of the magnitude, but not direction, of the Mendelian randomization estimate from instrumental variable analysis [34]. Instrumental variable analyses estimates generated as the association of genetic variant with the outcome divided by the association of genetic variant with the exposure may be inflated by reverse causality if the outcome affects the exposure in such a way as to reduce the association of the genetic variant with the exposure.

Our results suggest that moderate alcohol use has no effect on CVD, IHD or fasting glucose, which is different from western observational studies where moderate alcohol use is associated with lower morbidity or mortality from CVD and diabetes [1]–[3]. However, these findings from western settings have not always been replicated in non-western settings [6]–[8]. Such discrepancies might indicate that any observed protective effects in western contexts are due to confounding rather than to the biological properties of alcohol although the null results, in particular for CVD and IHD, had limited statistical power and do not preclude small protective effects. Alternatively, given that moderate alcohol use has both healthy and unhealthy effects on CVD risk factors the net effect may depend on the CVD risk profile in any given population [35]. Higher HDL-cholesterol is thought to underlie the cardioprotection of moderate alcohol use [36]. However, the causal role of HDL-cholesterol has been challenged by a recent meta regression analysis of RCTs of lipid modifying drugs and by two Mendelian randomization studies where genetically higher HDL-cholesterol had little effect on IHD [37]–[39]. The mechanistic pathway by which alcohol confers cardioprotection may need to be extended beyond HDL-cholesterol. Lower respiratory mortality has been observed with moderate alcohol use, with corresponding implications for CVD morbidity and mortality [40]. Respiratory benefits of alcohol may occur via anti-inflammatory pathways [41]. Alternatively, alcohol reduces testosterone [42], which would be expected to reduce inflammation [43], giving a possible pathway. However, to our knowledge whether alcohol operates via these pathways has not been comprehensively assessed.

From a public health perspective, our study using a potentially less biased design, suggests no benefit of moderate alcohol use on CVD, IHD or some CVD risk factors including fasting glucose. On the other hand, our null associations of moderate alcohol use with IHD and CVD might suggest a non-causal role of moderate alcohol use, but had limited statistical power. Larger studies are needed to confirm these findings.
